# Social environment, health cognition, and health behavior: how individuals with non-fixed employment end up with adverse health outcomes in China under the era of VUCA?—findings from PLS-SEM and fsQCA

**DOI:** 10.3389/fpubh.2024.1341213

**Published:** 2024-08-20

**Authors:** Haibin Wei, Qiaoqi Wang, Jianyang Chen, Zhenyi Liang, Yibo Wu, Hongye Luo

**Affiliations:** ^1^Guangxi University, Nanning, China; ^2^First Affiliated Hospital, Guangxi Medical University, Nanning, China; ^3^Chancellor’s Office, Guangxi Traditional Chinese Medical University, Nanning, China; ^4^School of Public Health and Management, Guangxi Traditional Chinese Medical University, Nanning, China; ^5^School of Public Health, Health Science Centre, Peking University, Beijing, China; ^6^School of Information and Management, Guangxi Medical University, Nanning, China

**Keywords:** social environment, health cognition, health behavior, non-fixed employment, adverse health outcomes, mediating mechanism, configurational analysis

## Abstract

**Objectives:**

This article studied the single-factor causal relationships between the social environment, health cognition, and health behavior of the individuals with non-fixed employment and their adverse health outcomes, as well as the complex causal relationships of multiple factors on these outcomes.

**Methods:**

Partial Least Squares Structural Equation Modeling (PLS-SEM) and Fuzzy-Set Qualitative Comparative Analysis (fsQCA) are employed. Data is collected from the results of an open questionnaire *Psychology and Behavior Investigation of Chinese Residents 2021.*

**Results:**

PLS-SEM analysis reveals that health risk behaviors and cognition play a mediating role in impact of the social environment on adverse health outcomes, indicating that individuals with non-fixed employment susceptible to adverse health outcomes. fsQCA analysis identifies that weak social support is a core condition leading to outcomes of depression and anxiety. There are shared configurations and causal pathways between the outcomes of physical health and depression.

**Conclusion:**

The study supports the social determinants theory of health and suggests that the fundamental reason for people being trapped in adverse health outcomes is the health inequality caused by social stratification, and the external shock of uncertainty in the era of VUCA (Volatility, Uncertainty, Complexity, and Ambiguity).

## Introduction

In a VUCA world, the population without fixed employment has been on the rise. The abbreviation “VUCA” stands for Volatility, Uncertainty, Complexity, and Ambiguity. This term originated in the military context of the United States in the 1990s, addressing the characteristics of the post-Cold War era, particularly in dealing with actions against terrorist organizations, which were perceived as more complex and uncertain than ever before. After the outbreak of the COVID-19 pandemic in 2020, instances of complexity and uncertainty have become more frequent, making the VUCA era a new normal (1). It is often expressed as “The only certainty is uncertainty in the external environment” and “Change is the only constant.” VUCA (Volatility, Uncertainty, Complexity, and Ambiguity) era has gained increased attention following the outbreak of the COVID-19 pandemic (2–4). Uncertainty is one of the defining characteristics of the VUCA era, prominently exemplified by measures of curbing the COVID-19 pandemic leading to rising unemployment rates (5) and potential long-term “scarring effects” on the labor market. As relief policies have been gradually phased out, more individuals face the looming threat of joblessness (6). In April 2022, survey data from 31 major cities in China indicated an urban unemployment rate of 6.7%, surpassing the highest point during the first wave of the pandemic in 2020 (5.9% in May 2020) (7). However, the unemployed population represents just the tip of the iceberg in terms of the labor market shocks in the VUCA era, with a more profound impact on those without fixed employment. The term “non-fixed employment” refers to the working-age population that have no stable jobs, including the jobless, unemployed, and underemployed (excluding students) (8). This group falls at the tenth stratum of contemporary Chinese society (9), characterized by a lack of organizational, economic, and cultural capital (10). Unemployment has become a common phenomenon and source of stress during economic crises. The status of having no fixed occupation not only affects individuals’ physical health (11) but also contributes to negative health behaviors such as smoking, heavy drinking (12), and poor mental health (13). Consequently, individuals without fixed employment often experience adverse health outcomes, exhibiting worse health conditions compared to the general population (14).

On 5 May 2023, the World Health Organization declared the “Public Health Emergency of International Concern” caused by the COVID-19 pandemic to be over (15). However, the VUCA era persists, and the population without fixed employment continues to grow, with adverse health outcomes remaining prevalent. Therefore, our study aims to investigate the influencing factors and underlying mechanisms of adverse health outcomes among individuals without fixed employment. This research endeavors to provide guidance and recommendations for proactively addressing health risks in the VUCA era.

## Literature review and hypotheses

### Literature review

Unemployment can have a profound impact on an individual’s physical health, mental health and other aspects of life. Firstly, there is a widespread agreement among academics that unemployment negatively affects an individual’s physical health. Compared to those in regular employment, unemployed people are at a higher risk of developing arthritis (16), committing suicide, and dying from other diseases (all illnesses excluding cancer and cardiovascular diseases) (17). Secondly, it is a common view among scholars that depression is a prevalent consequence of the mental health impact of unemployment (18). The longer the period of unemployment, the higher the chance of suffering from major depressive disorder (19). Lastly, numerous scholars also suggest that unemployment can lead to poor dietary habits (20) and increased alcohol consumption (21). Moreover, individuals with mental and physical health issues are at a higher risk of unemployment (22, 23). It is evident that unemployment can comprehensively affect an individual’s health status and can easily create a vicious cycle of mutual influence.

With the advancement of research, scholars have increasingly concentrated on the impact of unemployment on health. To this end, the academic community has primarily developed three representative viewpoints. The first is the perspective of lacking social support, which means that unemployment leads to reduced levels of social support and a decline in mental health (24). The second is the perspective of social exclusion, suggesting that unemployment often leads to social exclusion (25) and consequently a worsened psychosocial environment and health status (26). The third is the perspective of lack of health opportunities, arguing that unemployment results in fewer opportunities for medical care (27) and social resources (28), thereby impacting health levels. In essence, unemployment leads to a decrease in an individual’s socioeconomic status of an individual, which is then accompanied by a reduction in social support, health opportunities, and a lack of various resources, ultimately affecting the health of unemployed people.

How to mitigate the negative impact of unemployment on health. From a macro perspective, governments can issue unemployment allowances (29), social security, and welfare policies (30). However, some scholars have pointed out that the amount of unemployment relief is too low to significantly reduce men’s economic stress (31). And at the same time, differences in economic level and social resources can also lead to differences in health outcomes for unemployed people after receiving unemployment allowances (32). From a meso perspective, family and social support are very important for maintaining mental health during unemployment (33). Information, economic level or emotional support (34), strong social capital (the number of close friends) (35) helps to mitigate the negative impact of unemployment on health. In addition, there are also a few studies from a micro perspective that believe individual emotional intelligence (36) and leisure activities (37) can mitigate the negative impact of stress related to unemployment on mental health.

Based on the literature review, we believe that there are three research gaps. First, scientifically comprehensive measurement was conducted on adverse health outcomes as the dependent variable. Previous research has mainly focused on single dimensions of physiological health or mental health, lacking comprehensive measurement and explanation. This study integrated commonly used measures in the academic field, including the Chinese version of the European Quality of Life-5 Dimensions-5 Levels (EQ-5D-5 L), the Patient Health Questionnaire-9 (PHQ-9) for depression screening, and the Generalized Anxiety Disorder-7 (GAD-7) scale, to comprehensively measure physiological and mental health indicators, providing a scientific and comprehensive assessment of adverse health outcomes among individuals with non-fixed employment. Second, this study comprehensively considered both individual traits and external environment of individuals with non-fixed employment. Previous studies mainly focused on individual traits while neglecting the influence of the external environment. Given the externality of health outcomes, exploring their influencing factors requires consideration not only of individual traits but also of the external environment, including family environment and social environment. Third, this study utilized the advantages of quantitative research methods and qualitative comparative analysis. Previous studies primarily utilized quantitative methods to explore the influencing factors. They also explored linear relationships between influencing factors and adverse health outcomes among individuals with non-fixed employment. However, they failed to reveal the multiple causal relationships among numerous influencing factors and adverse health outcomes. This study integrates PLS-SEM and fsQCA, which not only explains the interactions between various influencing factors of individuals with non-fixed employment and adverse health outcomes but also reveals the multiple causal relationships among numerous influencing factors and adverse health outcomes.

### Hypotheses

Adverse health outcomes are primarily influenced by social support and family health. Social support may have indirect effects on health through enhanced mental health, or by reducing the impact of stress (38). Individuals with no social ties or very few social ties exhibit the most pronounced risk of poor health (39). Repeated experience of unemployment results in fewer social support for workers, thereby posing stress on their health (40). Additionally, social network is one of the key determinants of heath and deteriorating social networks constitute a major adverse consequence of unemployment (41). Furthermore, family plays a crucial role in affecting health directly, while intimate friends and family members can promote individual’s well-being (42). To this end, we propose the following hypotheses:

Hypothesis 1: Social environment has a negative impact on adverse health outcomes.

Hypothesis 1-1: Social support has a negative impact on adverse health outcomes.

Hypothesis 1-2: Family health has a negative impact on adverse health outcomes.

Individuals without fixed employment often possess lower health literacy (43). Due to their disadvantaged social environment, individuals with non-fixed employment struggle to obtain and understand accurate, high-quality health information (44). Therefore, we propose the following hypotheses:

Hypothesis 2: The social environment has a positive impact on health cognition.

Hypothesis 2-1: Social support has a positive impact on health cognition.

Hypothesis 2-2: Family health has a positive impact on health cognition.

Social support has been shown to be associated with various health behaviors and outcomes. Socioeconomic resources shape health outcomes by influencing health risk behaviors, access to healthcare, and exposure to stressful life events (45, 46). Unstable employment status is often accompanied by poorer self-rated health, health behaviors, and mental health (47). Meanwhile, social connections can influence health behavior habits, and emotional support provided by social relationships can enhance psychological well-being, thereby reducing unhealthy behaviors and health risks (38). Additionally, family members within the same family system exhibit similar lifestyle habits and norms, which can mutually influence each other (48). Therefore, we propose the following hypotheses:

Hypothesis 3: Social environment has a negative impact on health risk behaviors.

Hypothesis 3-1: Social support has a negative impact on health risk behaviors.

Hypothesis 3-2: Family health has a negative impact on health risk behaviors.

There exists a close association between the level of health literacy and health outcomes. Health literacy is a key determinant of health (49), and it has been suggested that persons with low health literacy suffer from poorer overall health (50). Moreover, one of the population groups with a particularly high risk for low health literacy is unemployed persons (51). According to Bandura’s Social Cognitive Theory, a lack of social support may exacerbate adverse health behaviors and conditions among individuals with low health literacy (52), and higher health literacy is conducive to improving adverse health outcomes (53). Therefore, we propose the following hypothesis:

Hypothesis 4: Health cognition has a negative impact on adverse health outcomes.

Adverse health outcomes are largely related to health risk behaviors. Smoking, heavy alcohol drinking, unhealthy diet, and lack of physical exercise are the main causes of adverse health outcomes (15). Individuals with low socioeconomic status, in relative deprivation, are more susceptible to unemployment (54). Unemployment, as a stress-inducing life event, exerts serious negative effects on an individual’s health and health behaviors (55). Smoking, excessive alcohol consumption, and binge eating are coping mechanisms against stress (56), contributing to the poor health status of long-term unemployed individuals (57). Therefore, we propose the following hypothesis:

Hypothesis 5: Health risk behaviors have a positive impact on adverse health outcomes.

Health literacy has a widespread influence on individual health outcomes. An individual’s health literacy directly determines oneself health behaviors (58). Individuals with lower health literacy may experience a higher the risk of medical errors due to misunderstandings of medical information (59). Since employment status directly affects personal income, healthcare accessibility, and social status, low health literacy among individuals with non-fixed employment can have a negative impact on their health (60). Therefore, we propose the following hypothesis:

Hypothesis 6: Health cognition mediates the impact of the social environment on adverse health outcomes.

Hypothesis 6-1: Health cognition mediates the impact of social support on adverse health outcomes.

Hypothesis 6-2: Health cognition mediates the impact of family health on adverse health outcomes.

Hypothesis 7: Health risk behaviors mediate the impact of the social environment on adverse health outcomes.

Hypothesis 7-1: Health risk behaviors mediate the impact of social support on adverse health outcomes.

Hypothesis 7-2: Health risk behaviors mediate the impact of family health on adverse health outcomes.

**Figure 1:**
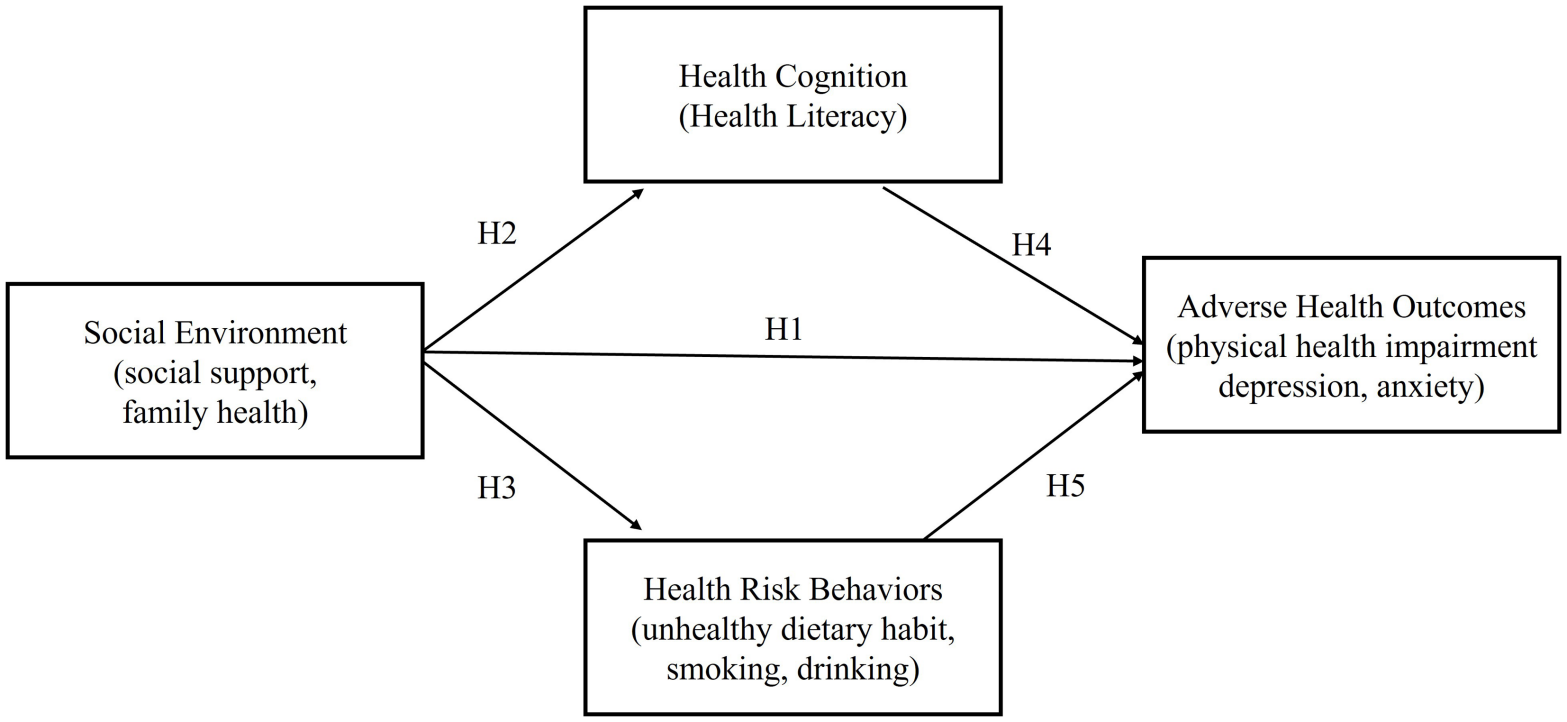
Research model.

Based on this, we construct a model for the formation mechanism of adverse health outcomes among the non-fixed employment population, as shown in Figure 1.

## Materials and methods

### Research design

Based on the results from the open questionnaire *Psychology and Behavior Investigation of Chinese Residents 2021* (PBICR-2021), the formation mechanisms of adverse health outcomes among individuals with Non-Fixed Employment were explored using PLS-SEM and fsQCA methods.

### Data source

The research data is derived from the PBICR-2021, which includes all provinces, autonomous regions, and municipalities directly under the central government. A total of 120 cities were selected using random number tables from non-provincial capital cities in each province and autonomous region. The population of each city was stratified by gender, age, and urban–rural distribution. Sample sizes for each stratum were determined based on the population characteristics from the results of the 2021 Seventh National Population Census. Surveyors conducted convenient sampling while meeting quota requirements (61), and obtained approval from the Biomedical Ethics Committee of Peking University (JNUKY-2021-018). The sample number of individuals with non-fixed employment is 2,195, including 953 males (43.4%) and 1,242 females (56.6%). The age distribution is as follows: 10 individuals aged 18 and below (0.5%), 302 individuals aged 19–30 (13.8%), 404 individuals aged 31–40 (18.4%), 732 individuals aged 41–50 (33.3%), and 747 individuals aged 51 and above (34.0%). In terms of education, 1,937 individuals had an associate degree or below (88.2%), 223 had a bachelor’s degree (10.2%), 20 had a master’s degree (0.9%), and 15 had a doctorate or higher (0.7%). Regarding monthly income, 455 individuals earned 1,500 yuan or less (20.7%), 1,089 earned between 1,501 and 4,500 yuan (49.6%), 399 earned between 4,501 and 7,500 yuan (18.2%), 156 earned between 7,501 and 10,500 yuan (7.1%), and 95 earned 10,501 yuan or more (4.4%). The urban population consists of 1,094 individuals (49.8%), while the rural population consists of 1,101 individuals (50.2%).

### Variable measurement

#### Dependent variables and its measurement

##### Adverse health outcomes

This section covers three dimensions: physical health impairment, depression, and anxiety. Physical health impairment is assessed using the Chinese version of EQ-5D-5L developed by Luo et al. (62). This scale measures an individual’s health-related quality of life and consists of 5 items. Each item is rated on a Likert five-point scale, ranging from “no problems” (1 point) to “extreme problems” (5 points). Scores are then converted using a utility value scoring system specifically designed for the Chinese population. Depression is measured using the Patient Health Questionnaire-9 (PHQ-9) developed by Kroenke et al. (63). This self-report tool is designed to screen for and assess the severity of depression symptoms based on the Diagnostic and Statistical Manual of Mental Disorders (DSM-IV) criteria published by the American Psychiatric Association. It consists of 9 items, each rated on a Likert four-point scale, with responses such as “not at all “, “several days,” “more than half the days,” and “nearly every day,” corresponding to scores of 0, 1, 2, and 3, respectively. A representative statement is that “Feeling down, depressed, or hopeless about everything?” Anxiety is assessed using the Generalized Anxiety Disorder-7 (GAD-7) scale developed by Spitzer et al. (64). This scale is suitable for screening generalized anxiety disorder and assessing symptom severity. It consists of 7 items, each rated on a Likert four-point scale, with responses similar to the PHQ-9. Scores range from 0 for “not at all” to 3 for “nearly every day.” A typical statement is “Feeling nervous, anxious, or on edge?”

#### Independent variables and its measurement

##### Social environment

It includes two dimensions: social support and family health. Social support is measured using the Perceived Social Support Scale (PSSS) introduced by Blumenthal (65) and developed by Zimet and the Chinese version translated by Jiang Qianjin is adopted in this research (66). The scale measures the extent of an individual’s social support, which includes three dimensions: family support, friend support, and other support, with a total of 12 items. Each item uses a Likert seven-point rating scale, ranging from “strongly disagree” (1 point) to “strongly agree” (7 points). An example statement is that “I can get emotional help and support from my family when I need it.” Family health is measured using the Family Health Scale-Short Form (FHS-SF) developed by Crandall et al. (67). The scale measures family health function with a total of 10 items. Each item uses a Likert five-point rating scale, ranging from “strongly disagree” (1 point) to “strongly agree” (5 points). An example statement is that “In my family, we support each other.”

##### Health cognition

This involves a single dimension, health literacy, and is measured using the Health Literacy Scale-Short Form12 (HLS-SF12) developed by Van Duong et al. (68). The scale measures an individual’s level of health literacy, including three dimensions: medical health, disease prevention, and health promotion. Each dimension involves four items related to mastery, understanding, judgment, and application, resulting in a total of 12 items. Each item uses a Likert four-point rating scale, ranging from “very difficult” (1 point) to “very easy” (4 points). Standardization is performed using the formula index score = (mean − 1) × (50/3). A representative statement is that “Find treatment information for your diseases.”

##### Health risk behaviors

This includes three dimensions: unhealthy dietary habits, smoking, and drinking. Unhealthy dietary habits are measured using the Eating Behavior Scale (EB) developed by Tayama et al. (69). The scale assesses an individual’s eating behavior with a total of 7 items. Each item uses a Likert four-point rating scale, ranging from “strongly disagree” (1 point) to “strongly agree” (4 points), with higher scores indicating worse eating behavior. A representative statement is that “I have no regular eating time.” Smoking and drinking are single-item measures, with scoring based on the number of cigarettes smoked and the frequency of drinking set by Xia Delong et al. (70). Smoking is scored as follows: never smoking = 1, 1–5 cigarettes = 2, 6–10 cigarettes = 3, 11–20 cigarettes = 4, more than 20 cigarettes = 5. A higher score indicates more smoking. Drinking frequency is scored as follows: 0 times = 1, an average of 1–2 times per week = 2, an average of 3–4 times per week = 3, almost every day = 4. A higher score indicates higher drinking frequency. Self-rated health is one of the most common measurements of global health in social science and public health research and has been demonstrated to have high validity (71).

### Control variables

The study selects gender, age, education level, *per capita* monthly income, and place of residence as control variables.

### Research method selection

#### Partial least squares structural equation modeling

Hair et al. (72) argued that partial least squares structural equation modeling (PLS-SEM) is more suitable for complex models due to its stronger predictive capabilities compared to covariance-based SEM. It is also suitable for exploring or extending theoretical models. Therefore, SPSS 26.0 and SmartPLS 4 were used for statistical analysis of the data. This involved four steps. First, the Harman’s one-factor test was used to check for common method bias (73). Second, following Hair et al.’s (72) rules-of-thumb for reflective measurement models assessment, the internal consistency reliability (Cronbach’s alpha, Composite Reliability), convergent validity (Outer Loadings, AVE), and discriminant validity (cross-loadings, HTMT, Fornell-Larcker criterion) of the measurement model were assessed. Third, based on Hair’s rules-of-thumb (72) for evaluating structural models, assessments were made regarding multicollinearity among predictor variables, the significance of path coefficients using bootstrap method, the determination coefficient R^2^ of endogenous latent variables, the relative contribution of exogenous constructs to the determination coefficient of endogenous latent variables (effect size f^2^), predictive relevance (Stone-Geisser Q^2^), and model fit indices (SRMR). Fourth, Baron and Kenny (74)‘s four-step approach was referred to test the significance of mediating effect, and further, the Bootstrapping method using SmartPLS software was conducted to generate 5,000 iterations to estimate confidence intervals of mediating effect.

#### Fuzzy-set qualitative comparative analysis

PLS-SEM has limitations in explaining complex interaction effects, especially those involving three or more influencing factors (75). Qualitative Comparative Analysis (QCA) offers a suitable means to accommodate complex complementarity and nonlinear relationships among variables (75, 76). QCA takes research cases as configurations of conditions or attributes (77). Fuzzy-set qualitative comparative analysis (fsQCA) allows for a more detailed exploration of the possible configurations that lead to specific outcomes and is advantageous for in-depth discovery of conditions that contribute to the occurrence of results (78). This method involves four steps. First, data calibration. Second, construction of truth table. Third, necessity analysis. Fourth, configuration analysis. Therefore, social support, family health, health literacy, unhealthy eating behavior, smoking, and drinking are selected as antecedent conditions of adverse health outcomes among individuals without fixed employment, so as to further elucidate the mechanism behind adverse health outcomes in this population group.

## PLS-SEM results

### Common method bias

To test for common method bias, the Harman one-factor test was employed. All items from the measurement scales used in this study were subjected to factor analysis. Through principal component analysis, the variance contribution rate of first unrotated factor was found to be 28.875%, which is less than 50% (79). This indicates the absence of significant common method bias.

### Measurement model assessment

Measurement model consists of reflective measurement model and formative measurement model, and the one employed in the study is reflective measurement model. The internal consistency of the models is presented in Table 1. The Cronbach’s alpha coefficients for all measurement variables are greater than 0.7, and all the Composite Reliability (CR) values are higher than 0.7 (with a minimum value of 0.794), indicating strong internal consistency of the measurement model. Convergent validity is shown in Table 1, the outer loadings of each measurement model were greater than 0.7, and the Average Variance Extracted (AVE) values were all above 0.5 (with a minimum value of 0.564), indicating good convergent validity of the measurement models.

**Table 1:** Reliability and validity.

	Cronbach’s alpha	CR	AVE
Family support	0.952	0.950	0.658
Social support	0.938	0.958	0.656
Social environment	0.953	0.873	0.774
Health cognition	0.930	0.940	0.568
Health risk behaviors	0.854	0.794	0.564
Adverse health outcomes	0.944	0.852	0.658

Discriminant validity (cross loadings, HTMT, Fornell-Larcker criterion) was also evaluated. First, the evaluation standard for cross loadings of factors of each measurement model is that an indicator’s external loading on its own construct should be higher than its cross loadings with all the other constructs. In this study, all indicators effectively distinguished their respective constructs. Second, as shown in Table 2, the criterion for Heterotrait-Monotrait Ratio (HTMT) states that the HTMT values between constructs should not exceed 0.85, and the confidence intervals for HTMT statistics should not include 1. Finally, as indicated in Table 3, discriminant validity was tested using the Fornell-Larcker criterion (80). Upon comparison, the square root of AVE values for each variable are higher than the correlation coefficients between variables. The results demonstrated good discriminant validity among the variables in this study.

**Table 2:** HTMT-based discriminant validity evaluation.

	1	2	3	4	5
1. Adverse health outcomes					
2. Health cognition	0.470				
3. Health risk behaviors	0.563	0.642			
4. Family health	0.428	0.313	0.269		
5. Social support	0.487	0.368	0.364	0.582	

**Table 3:** Relevant analysis and Fornell-Larcker criterion.

	Mean	Std. Deviation	1	2	3	4	5	6
1. Social support	57.983	12.570	0.811					
2. Family health	36.862	8.150	0.550^**^	0.810				
3. Social environment	94.845	18.363	-	-	0.880			
4. Health cognition	34.579	6.154	0.345^**^	0.291^**^	0.365^**^	0.753		
5. Health Risk behaviors	1.697	0.538	−0.277^**^	−0.200^**^	−0.278^**^	−0.493^**^	0.751	
6. Adverse health outcomes	17.277	10.434	−0.414^**^	−0.363^**^	−0.445^**^	−0.376^**^	0.341^**^	0.811

^**^The square root of AVE value is higher than the correlation coefficients between variables.

### Structural model evaluation

**Figure 2:**
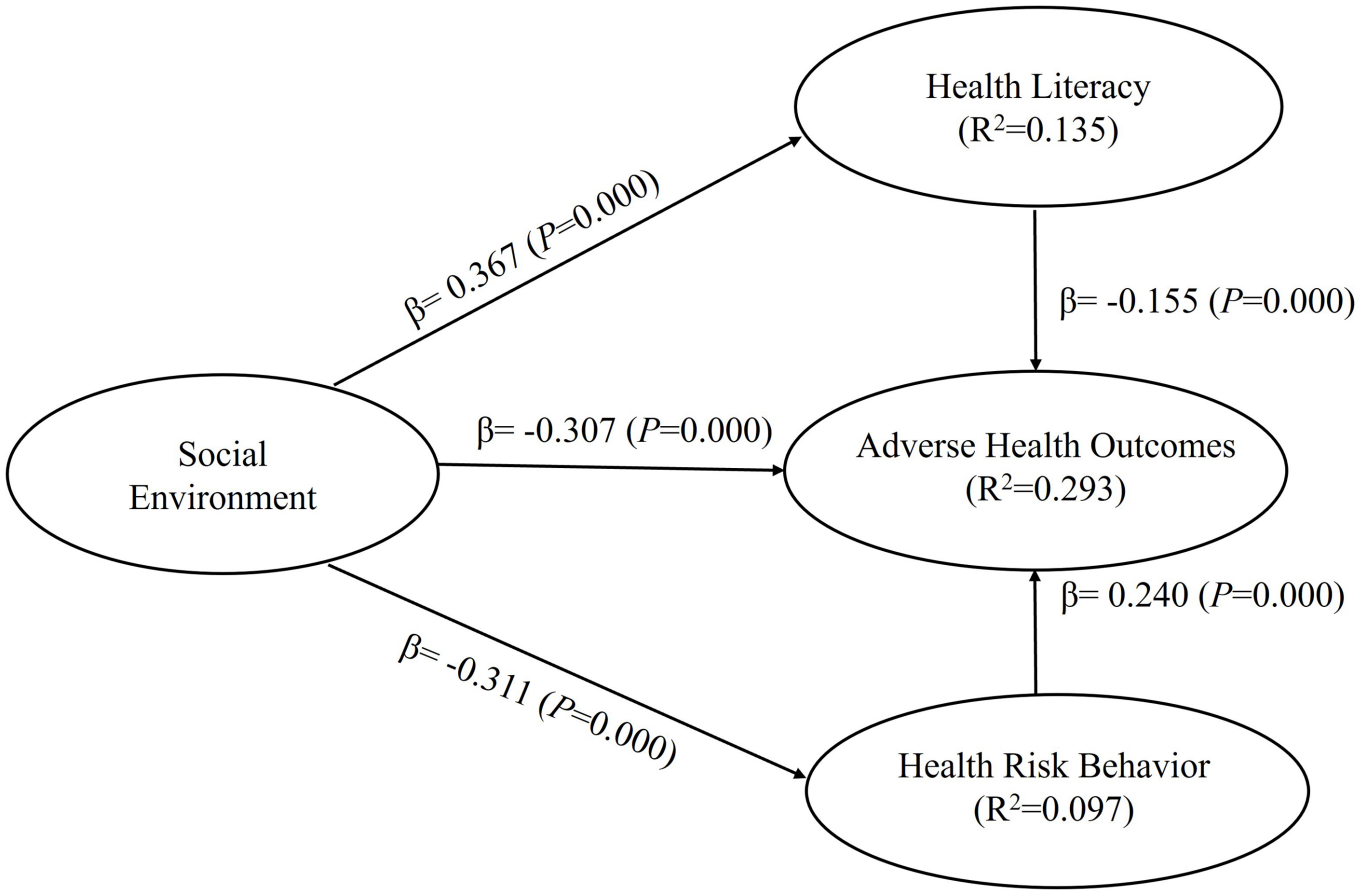
Results of structural model 1.

**Figure 3:**
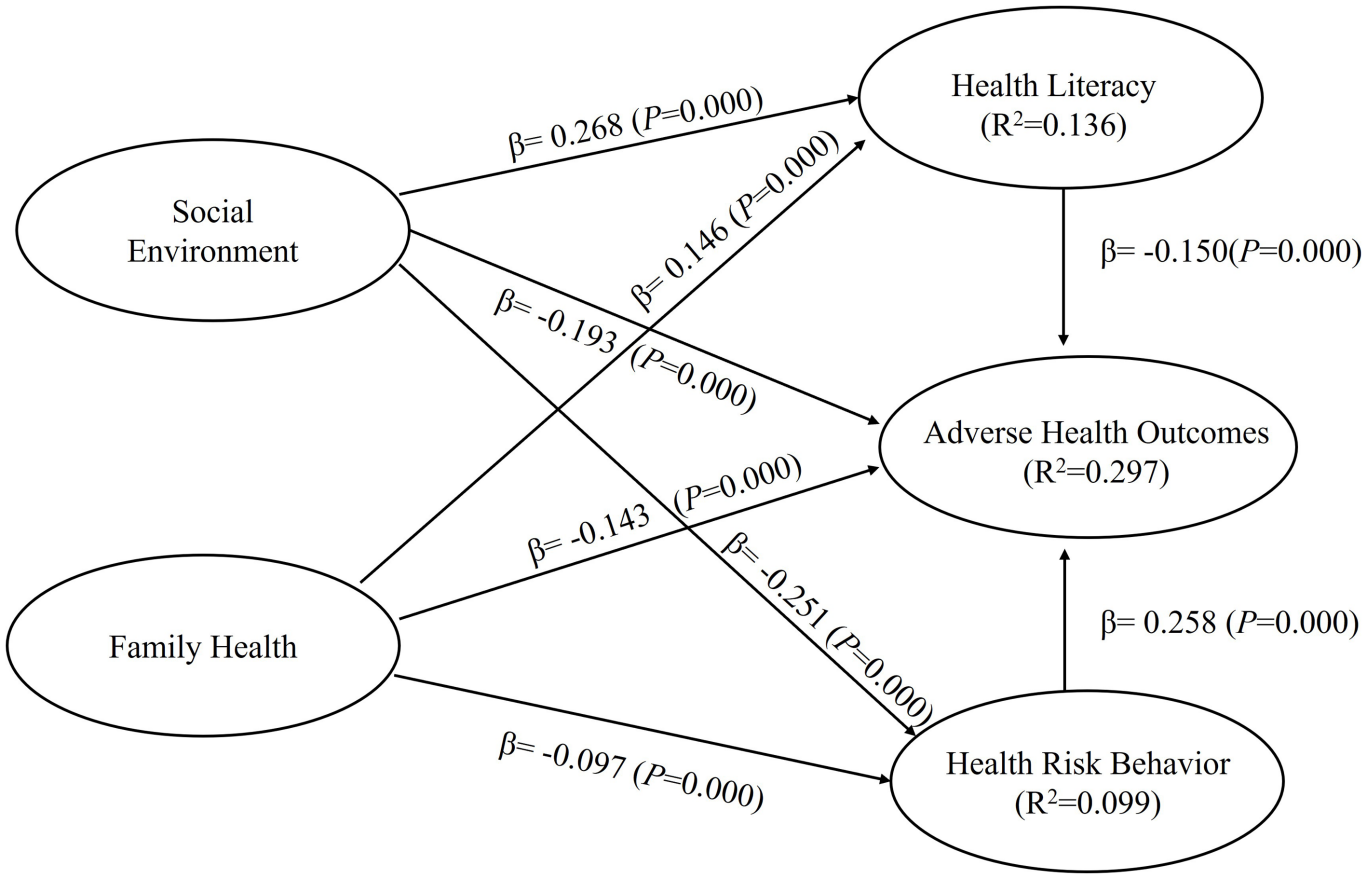
Results of structural model 2.

Based on the research hypotheses, two mediation models were constructed. Model 1 employed social environment as the independent variable, with health cognition and health risk behavior as the mediating variables, and adverse health outcomes as the dependent variable, creating a dual-path mediation model (as depicted in Figure 2). To separately investigate the effects of social support, the subdimensions of social environment, and family health on adverse health outcomes, Model 2 was constructed. In this model, social support and family health served as independent variables, while health cognition and health risk behavior acted as mediating variables, and adverse health outcomes remained the dependent variable, creating a dual-path mediation model (as shown in Figure 3). The results of the model tests are presented in Table 4.

**Table 4:** Results of model tests.

Hypotheses		Std. estimate	S.E.	t	P	f^2^	VIF	Decision
Mediation Model 1								
H1	Social environment → Adverse health outcomes	−0.307	0.029	10.586	0.000	0.112	1.184	Support
H2	Social environment → Health cognition	0.367	0.024	15.017	0.000	0.156	1.000	Support
H3	Social environment → Health risk behaviors	−0.311	0.020	15.557	0.000	0.107	1.000	Support
H4	Health cognition → Adverse health outcomes	−0.155	0.028	5.549	0.000	0.024	1.440	Support
H5	Health risk behaviors → Adverse health outcomes	0.240	0.027	8.810	0.000	0.059	1.379	Support
Mediation Model 2								
H1-1	Social support → Adverse health outcomes	−0.193	0.030	6.539	0.000	0.034	1.545	Support
H1-2	Family health → Adverse health outcomes	−0.143	0.026	5.602	0.000	0.020	1.465	Support
H2-1	Social support → Health cognition	0.268	0.026	10.297	0.000	0.058	1.438	Support
H2-2	Family health → Health cognition	0.146	0.025	5.837	0.000	0.017	1.465	Support
H3-1	Social support → Health risk behaviors	−0.251	0.025	10.142	0.000	0.049	1.438	Support
H3-2	Family health → Health risk behaviors	−0.097	0.026	3.655	0.000	0.007	1.438	Support

First, in Model 1, the maximum Variance Inflation Factor (VIF) value for all predictor variables was 1.440. In Model 2, the maximum VIF value for all predictor variables was 1.545. Both values were less than 5, indicating no significant multicollinearity among the constructs.

Second, after applying the Bootstrapping method and performing 5,000 resamples to the mediation models, the analysis results of Model 1 showed that social environment had a significant negative effect on adverse health outcomes (β = −0.307, 95% CI = [−0.361, −0.248]), supporting Hypothesis H1. Social environment also had a significant positive effect on health cognition (β = 0.367, 95% CI = [0.317, 0.414]) and a significant negative effect on health risk behavior (β = −0.311, 95% CI = [−0.350, −0.271]), supporting Hypotheses H2 and H3, respectively. Health cognition had a significant negative effect on adverse health outcomes (β = −0.155, 95% CI = [−0.208, −0.097]), supporting Hypothesis H4, and health risk behavior had a significant positive effect on adverse health outcomes (β = 0.240, 95% CI = [0.187, 0.294]), supporting Hypothesis H5. In Model 2, the analysis results showed that social support had a significant negative effect on adverse health outcomes (β = −0.193, 95% CI = [−0.248, −0.132]), supporting Hypothesis H1-1. Family health also had a significant negative effect on adverse health outcomes (β = −0.143, 95% CI = [−0.193, −0.091]), supporting Hypothesis H1-2. Social support had a significant positive effect on health cognition (β = 0.268, 95% CI = [0.215, 0.316]), supporting Hypothesis H2-1, and family health had a significant positive effect on health cognition (β = 0.146, 95% CI = [0.098, 0.196]), supporting Hypothesis H2-2. Social support had a significant negative effect on health risk behavior (β = −0.251, 95% CI = [−0.297, −0.199]), supporting Hypothesis H3-1, and family health had a significant negative effect on health risk behavior (β = −0.097, 95% CI = [−0.149, −0.045]), supporting Hypothesis H3-2. Health cognition had a significant negative effect on adverse health outcomes, and health risk behavior had a significant positive effect on adverse health outcomes, confirming Hypotheses H4 and H5, respectively.

Third, the coefficient of determination (R^2^) is a measure of the model’s predictive ability. It is calculated by squaring the correlation between the actual values and predicted values of specific endogenous structures, indicating the overall impact of exogenous latent variables on endogenous latent variables. In Model 1, the R^2^ values for health cognition, health risk behavior, and adverse health outcomes of the endogenous latent variables were 0.135, 0.097, and 0.293, respectively. In Model 2, the R^2^ values for health cognition, health risk behavior, and adverse health outcomes of the endogenous latent variables were 0.136, 0.099, and 0.297, respectively. According to rules of thumb, R^2^ values of 0.75, 0.50, or 0.25 of the endogenous latent variables represent strong, moderate, or weak effects, respectively.

Fourth, the effect size ƒ^2^ measures the change in R^2^ when specific exogenous constructs are omitted from the model, providing an assessment of whether the omitted exogenous constructs have a substantial impact on the endogenous constructs. In Model 1, the ƒ^2^ for each path ranged from 0.024 to 0.156, while in Model 2, the ƒ^2^ for each path ranged from 0.007 to 0.058. The guidelines for evaluating ƒ^2^ suggest that values of 0.02, 0.15, and 0.35 represent small, medium, and large effects of exogenous latent variables, respectively, with values less than 0.02 indicating no effect.

Fifth, the Stone-Geisser Q^2^ for predictive relevance is obtained by using the blindfolding procedure on a specified omission distance D. Blindfolding is a sample re-use technique, which omits every D-th data point in the indicators of endogenous constructs, and estimates parameters by using the remaining data points. The omitted data represent missing values and are treated accordingly (e.g., by pairwise deletion or mean value replacement). The results then are used to predict the omitted data points. The difference between the omitted data points and the predicted ones is used to calculate the Q^2^ value. In Model 1, the Stone-Geisser Q^2^ values for social environment, health cognition, health risk behavior, and adverse health outcomes were 0.302, 0.487, 0.167, and 0.317, respectively. In Model 2, the Stone-Geisser Q^2^ values for social support, family health, health cognition, health risk behavior, and adverse health outcomes were 0.567, 0.584, 0.485, 0.154, and 0.243, respectively. A Q^2^ value larger than zero for a certain endogenous latent variable indicates the model has predictive relevance for the construct, while values at or below 0 indicate a lack of predictive relevance.

Sixth, PLS-SEM is based on the variance-based structural equation modeling (VB-SEM) principles. As a variance-based algorithm, it aims to maximize the explanatory power of endogenous variables. It differs from covariance-based SEM (CB-SEM), which is based on the covariance matrix and seeks to make the covariance matrix of samples as close as possible to the expected covariance matrix of the model, minimizing the residuals (squared differences). Consequently, global fit indices used in CB-SEM are not suitable for PLS-SEM. Henseler et al. (81) have suggested the SRMR (Standardized Root Mean Square Residual) as fit indices, with standards of less than 0.08 (ideal) or 0.12 (acceptable). In Model 1, the SRMR was 0.110, while in Model 2, the SRMR was 0.066.

### Mediation effects assessment

For the mediation effects of health cognition and health risk behavior in the relationships between the social environment and its dimensions (family health and social support) and adverse health outcomes, the Bootstrapping method was conducted (5,000 resamples), and the results are presented in Table 5.

**Table 5:** Mediation effects assessment.

Hypothesis		Std. estimate	S.E.	Percentile Bootstrap		Decision
				95%LCI	95%UCI	
Mediation Model 1 (Social environment → Adverse health outcomes)						
	Total effect	−0.438	0.026	−0.486	−0.384	
	Direct effect	−0.307	0.029	−0.361	−0.248	
	Total indirect effect	−0.131	0.011	−0.154	−0.111	
H6	Social environment → Health cognition → Adverse health outcomes	−0.057	0.011	0.011	−0.036	Support
H7	Social environment →Health risk behaviors→ Adverse health outcomes	−0.074	0.009	0.009	−0.058	Support
Mediation Model 2 (Social Support/Family health→ Adverse health outcomes)						
	Social support→ adverse health outcomes					
	Total effect	−0.298	0.026	−0.353	−0.251	
	Direct effect	−0.193	0.027	−0.254	−0.148	
	Total indirect effect	−0.105	0.011	−0.124	−0.081	
H6-1	Social support → Health cognition → Adverse health outcomes	−0.040	0.009	−0.058	−0.024	Support
H7-1	Social support → Health risk behaviors → Adverse health outcomes	−0.065	0.010	−0.087	−0.047	Support
	Family health→ Adverse health outcomes					
	Total effect	−0.190	0.026	−0.243	−0.142	
	Direct effect	−0.143	0.024	−0.193	−0.099	
	Total indirect effect	−0.047	0.009	−0.064	−0.029	
H6-2	Family health →Health cognition → Adverse health outcomes	−0.022	0.006	−0.033	−0.012	Support
H7-2	Family health → Health risk behavior s → Adverse health outcomes	−0.025	0.007	−0.041	−0.012	Support

Regarding the mediating effect of health cognition on the relationship between the social environment and adverse health outcomes, the 95% confidence interval was [−0.079, −0.036] with the Bootstrapping method. Since this confidence interval does not include zero, it indicates a significant mediating effect of health cognition between the social environment and adverse health outcomes. The standardized effect size was −0.057, supporting the hypothesis H6. Similarly, for the mediating effect of health risk behavior on the relationship between the social environment and adverse health outcomes, the Bootstrapping method yielded a 95% confidence interval of [−0.093, −0.058], with a β value of −0.074, supporting hypothesis H7.

Concerning the mediating effects of health cognition and health risk behavior on the relationships between social support, family health, and adverse health outcomes, the results are as follows. Health cognition mediates the relationship between social support and adverse health outcomes, with a 95% confidence interval of [−0.058, −0.024] and a β value of −0.040, supporting hypothesis H6-1. Health cognition also mediates the relationship between family health and adverse health outcomes, with a 95% confidence interval of [−0.033, −0.012] and a β value of −0.022, supporting hypothesis H6-2. Health risk behavior mediates the relationship between social support and adverse health outcomes, with a 95% confidence interval of [−0.087, −0.047] and a β value of −0.065, supporting hypothesis H7-1. Health risk behavior mediates the relationship between family health and adverse health outcomes, with a 95% confidence interval of [−0.041, −0.012] and a β value of −0.025, supporting hypothesis H7-2.

## fsQCA results

### Data calibration

The variables involved in the study were calibrated. Based on the scoring rules, the degrees of related antecedent conditions are defined as follows. No depression (0–4 points) was assigned a value of 1; mild depression (5–9 points) was assigned a value of 2; moderate depression (10–14 points) was assigned a value of 3; moderately severe depression (15–19 points) was assigned a value of 4; severe depression (20 points and above) was assigned a value of 5. No anxiety (0–4 points) was assigned a value of 1; mild anxiety (5–9 points) was assigned a value of 2; moderate anxiety (10–14 points) was assigned a value of 3; severe anxiety (15 points and above) was assigned a value of 4. Low social support status (12–36 points) was assigned a value of 1; moderate social support status (37–60 points) was assigned a value of 2; high social support status (61–84 points) was assigned a value of 3. Poor family health condition (<6 points) was assigned a value of 1; moderate family health condition (6–8 points) was assigned a value of 2; good family health condition (9 or 10 points) was assigned a value of 3. Additionally, the degree of physical health impairment, health literacy level, and unhealthy eating behavior were calibrated based on Ragin’s criteria of 5% (Fully Out), 95% (Fully In), and the crossover point of 50% (82). Furthermore, smoking and drinking were assigned as binary variables (0 for non-smoking and non-drinking, 1 for smoking and drinking). Specific details are provided in the tables.

### Construction of truth table and necessity analysis

Following Ragin’s approach, the algorithm function of truth table in the fsQCA software was used to compute the truth table with a result variable of 1 and no contradictory configurations. Necessity analysis was performed for individual antecedent conditions. Consistency and coverage are indicators of confirmed causal relationships in QCA analysis. Table 6 shows that the consistency and coverage of the antecedent conditions did not reach 0.9 or higher, which suggests that the occurrence of adverse health outcomes (physical health impairment, depression, anxiety) cannot be determined and explained by individual antecedent conditions alone. It is necessary to conduct configuration analysis of antecedent conditions.

**Table 6:** Results of necessity analysis of adverse health outcomes.

Conditions	Physical health impairment		Conditions	Depression		Conditions	Anxiety	
	Consistency	Coverage		Consistency	Coverage		Consistency	Coverage
PS	0.768	0.852	PS	0.851	0.285	PS	0.829	0.396
FH	0.561	0.854	FH	0.528	0.243	FH	0.485	0.318
HL	0.698	0.894	HL	0.728	0.282	HL	0.704	0.389
EB	0.510	0.766	EB	0.843	0.383	EB	0.803	0.520
SM	0.135	0.563	SM	0.216	0.273	SM	0.213	0.384
DW	0.280	0.602	DW	0.427	0.278	DW	0.421	0.390
~PS	0.384	0.829	~PS	0.729	0.475	~PS	0.708	0.658
~FH	0.535	0.755	~FH	0.800	0.341	~FH	0.798	0.485
~HL	0.457	0.781	~HL	0.829	0.429	~HL	0.740	0.545
~EB	0.628	0.897	~EB	0.664	0.287	~EB	0.600	0.370
~SM	0.865	0.768	~SM	0.784	0.210	~SM	0.787	0.301
~DW	0.720	0.799	~DW	0.573	0.192	~DW	0.579	0.277

The above information is compiled by the authors based on the calculation results of fsQCA software. PS, Social Support; FH, Family Health; HL, Family Literacy; EB, Unhealthy Eating Behaviors; SM, Smoking; DW, Drinking.

### Configuration analysis

In the configuration analysis, three types of solutions are reported: complex solutions, intermediate solutions, and parsimonious solutions (83). Many studies tend to favor intermediate solutions because they are closer to theoretical reality without becoming overly complex (84). Therefore, we will analyze the intermediate solutions for each of the adverse health outcomes: physical health impairment, depression, and anxiety.

#### Configuration and causal explanatory pathways for physical health impairment outcome

The configuration analysis results for individuals without a fixed occupation experiencing physical health impairment include 10 configurations of intermediate solution, as shown in Table 7. Social support, health literacy, unhealthy eating behavior, drinking, and smoking simultaneously appear in both parsimonious and intermediate solutions, representing core conditions for physical health impairment. The remaining conditions only appear in intermediate solutions but not in parsimonious solutions, indicating that they are peripheral conditions for physical health impairment. Through Boolean minimization, the pathway can be expressed as PS*~FH*~HL*~EB*~SM*~DW*. This pathway indicates that even if individuals have strong social support, infrequent unhealthy eating behavior, do not smoke or drink, they will still experience physical health impairment if their family health level is low, and they have low health literacy, and no fixed occupation.

**Table 7:** Configurations of antecedent conditions for physical health impairment.

Antecedent conditions	Configurations									
	EQ_1_	EQ_2_	EQ_3_	EQ_4_	EQ_5_	EQ_6_	EQ_7_	EQ_8_	EQ_9_	EQ_10_
Social support PS	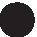	—	—	—	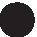	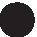	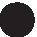	—	—	—
Family health FH	—	—	⊗	⊗	—	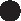	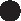	—	⊗	⊗
Family literacy HL	—	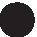	—	—	⊗	—	—	⊗	—	⊗
Unhealthy eating behaviors EB	—	—	⊗	⊗	⊗	—	⊗	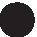	—	—
Smoking SM	⊗	⊗	—	⊗	—	—	—	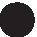	⊗	—
Drinking DW	—	—	⊗	—	—	⊗	—	⊗	⊗	⊗
Original coverage	0.672	0.624	0.250	0.300	0.326	0.374	0.394	0.018	0.342	0.196
Only coverage	0.037	0.042	0.002	0.004	0.011	0.001	0.002	0.001	0.034	0.000
Consistency	0.880	0.910	0.919	0.910	0.925	0.927	0.938	0.748	0.832	0.871
Overall consistency	0.862									
Overall coverage	0.840									

a. 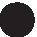 or 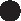 represents the presence of this condition in the configuration, ⊗ or ⊗ represents the absence of this condition, —Indicates that this condition is optional; b. 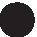 or ⊗ represents core conditions, 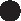 or ⊗ represents auxiliary conditions. EQ, EuroQol Five Dimensions Questionnaire.

#### Configuration and causal explanatory pathways for depression outcome

The configuration analysis results for individuals without a fixed occupation experiencing depression include 10 intermediate solution configurations, as shown in Table 8. The configuration analysis results for depression are consistent with those for physical health impairment, suggesting the possibility of shared causal pathways for both physical and mental adverse health outcomes in individuals with non-fixed employment. However, in the configuration for depression, the condition of social support appears in both parsimonious and intermediate solutions, indicating that it is a core condition for depression. The other conditions only appear in intermediate solutions but not in parsimonious solutions, suggesting that they are peripheral conditions for depression. After Boolean minimization, the pathway can be expressed as PS*~FH*~HL*~EB*~SM*~DW*. This pathway indicates that even if individuals have strong social support, infrequent unhealthy eating behavior, do not smoke or drink, they will still experience depression if their family health level is low, and they have low health literacy, and no fixed occupation. This causal pathway is consistent with that of physical health impairment.

**Table 8:** Configurations of antecedent conditions for depression.

Antecedent conditions	Configurations									
	PH_1_	PH_2_	PH_3_	PH_4_	PH_5_	PH_6_	PH_7_	PH _8_	PH _9_	PH _10_
Social support PS	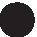	—	—	—	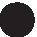	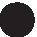	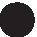	—	—	—
Family health FH	—	—	⊗	⊗	—	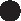	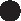	—	⊗	⊗
Family literacy HL	—	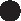	—	—	⊗	—	—	⊗	—	⊗
Unhealthy eating behaviors EB	—	—	⊗	⊗	⊗	—	⊗	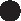	—	—
Smoking SM	⊗	⊗	—	⊗	—	—	—	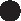	⊗	—
Drinking DW	—	—	⊗	—	—	⊗	—	⊗	⊗	⊗
Original coverage	0.676	0.614	0.339	0.451	0.567	0.312	0.415	0.039	0.419	0.377
Only coverage	0.035	0.010	0.000	0.006	0.025	0.001	0.001	0.001	0.014	0.000
Consistency	0.268	0.271	0.377	0.414	0.487	0.235	0.299	0.499	0.308	0.508
Overall consistency	0.865									
Overall coverage	0.255									

a. 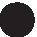 or 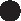 represents the presence of this condition in the configuration, ⊗ or ⊗ represents the absence of this condition, —Indicates that this condition is optional; b. 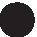 or ⊗ represents core conditions, 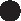 or ⊗ represents auxiliary conditions. PH, Patient Health Questionnaire.

#### Configuration and causal explanatory pathways for anxiety outcome

The configuration analysis results for individuals without a fixed occupation experiencing anxiety include two intermediate solution configurations, as shown in Table 9. Social support, family health, and health literacy simultaneously appear in both parsimonious and intermediate solutions, representing core conditions for anxiety. The remaining conditions only appear in intermediate solutions but not in parsimonious solutions, indicating that they are peripheral conditions for anxiety. After Boolean minimization, the pathway can be expressed as ~PS*~HL*EB. This pathway suggests that weak social support, low health literacy, and high-frequency unhealthy eating behavior will lead to anxiety outcomes in individuals without a fixed occupation.

**Table 9:** Configurations of antecedent conditions for anxiety.

Antecedent conditions	Configurations	
	GA_1_	GA_2_
Social support PS	⊗	⊗
Family health FH	⊗	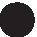
Family literacy HL	⊗	⊗
Unhealthy eating behaviors EB	—	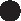
Smoking SM	⊗	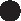
Drinking DW	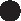	⊗
Original coverage	0.132	0.014
Only coverage	0.132	0.014
Consistency	0.796	0.831
Overall consistency	0.799	
Overall coverage	0.146	

a. 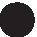 or 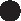 represents the presence of this condition in the configuration, ⊗ or ⊗ represents the absence of this condition, —Indicates that this condition is optional; b. 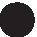 or ⊗ represents core conditions, 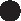 or ⊗ represents auxiliary conditions. GA, Generalized anxiety.

## Discussion

### Challenges for individuals with non-fixed employment in escaping external environmental control

Social factors play a crucial role in determining health outcomes, and an individual’s health status is closely linked to their social environment. Our study has demonstrated that the social environment has a significant negative impact, to the extent of 0.307%, on adverse health outcomes, with social support contributing negatively by 0.193% and family health by 0.143%. Furthermore, weak social support (~PS) has been identified as a core factor leading to depression and anxiety, as evidenced in configurations GA_1_ and GA_2_. This finding provides new evidence that aligns, to some extent, with Amartya Sen’s Capability Approach. The health outcomes of individuals with unstable employment depend on external social and family environment, while the external social and family environment also have considerable influence on their health outcomes. This aligns with the viewpoint put forth by Amartya Sen (85), who believes that health is a critical human “capability” that is often shaped by the social networks and social capital one possesses. Michael Marmot (86) has also asserted that the root cause of health inequalities lies in social inequality, with social determinants being the “causes of the causes” of health inequalities. When viewed from a family perspective, family not only significantly influences an individual’s health status over their entire lifespan but also impacts the health of multiple generations within the family (87). Therefore, individuals with non-fixed employment find it challenging to break free from external environmental control, and improving both the social and family environments is crucial for avoiding adverse health outcomes.

### Challenges for individuals with non-fixed employment in enhancing personal health cognition

Health cognition plays a pivotal role in influencing health outcomes, as an individual’s level of health literacy may determine their health outcomes. Our research has revealed that health cognition has a negative impact, to the extent of 0.155%, on adverse health outcomes. Meanwhile, low level of health literacy (~HL) has been identified as a core factor leading to anxiety, as confirmed in configurations GA_1_ and GA_2_. Moreover, low level of health literacy (~HL) also appears in the causal pathways of physical health impairment and depression, as reflected in configurations PH_5_, PH_8_, PH_10_, EQ_5_, EQ_8_, and EQ_10_. This finding, to a certain extent, supports the health communication theory. Health literacy is a direct manifestation of health cognition and can positively alter adverse health outcomes. However, individuals with unstable employment often face challenges in obtaining health education due to unfavorable living environments and limited access to social networks, resulting in lower health literacy (88). Therefore, it is crucial to focus on and improve the health literacy of individuals with unstable employment, provide health education, and disseminate health information to help them avoid adverse health outcomes.

### Challenges for individuals with non-fixed employment in changing health behaviors

Health behaviors have a significant impact on health outcomes, as the choices individuals make regarding their health behaviors can directly influence their health outcomes. Our research has confirmed that health risk behaviors have a substantial positive impact, amounting to 0.240%, on adverse health outcomes. Additionally, poor eating habits have been identified in the causal pathways leading to anxiety. Furthermore, unhealthy dietary habits, smoking, and alcohol consumption appear in configurations EQ_8_, PH_8_, GA_1_, and GA _2_, respectively. This finding provides new evidence that aligns with health behavior theory. Health behavior theory mainly explores the health-related consequences of health behaviors and influencing factors. It also holds that differences in health status among individuals are mainly attributed to variations in health behaviors (89). Smoking, drinking, and other unhealthy behaviors not only lead to adverse health outcomes but also significantly increase mortality rates (90). Moreover, the link between health behaviors and health outcomes is influenced by behavioral motivation (91), and behavioral motivation, along with health behaviors themselves, exhibits stability over time (92). Therefore, individuals with unstable employment face challenges in changing their health behaviors within a short period. It is crucial to focus on the health behaviors of individuals with non-fixed employment and improve their lifestyle habits to help them avoid adverse health outcomes.

### Challenge for individuals with non-fixed employment in escaping the trap of adverse health outcomes

Individuals with non-fixed employment often find themselves in unfavorable social environment, leading to lower levels of health cognition, ultimately resulting in adverse health outcomes. Our research has confirmed that health cognition plays a mediating role of −0.057% in the influence of the social environment on adverse health outcomes. Specifically, health cognition has a mediating effect of −0.040% of social support on adverse health outcomes and −0.022% of family health on adverse health outcomes. As the shared configurations and causal pathways for both physical health impairment and depression outcomes shown in the fsQCA analysis, adverse physical and mental health outcomes among unstable employment populations result from the combined effects of multiple antecedent conditions, and these adverse health outcomes may have relations with each other. This, to a certain extent, provides new evidence for social cognitive theory. We have extended and refined this theory by focusing on adverse health outcomes as a more realistic and theoretically valuable dependent variable.

Individuals with non-fixed employment, due to their exposure to harsher social environment, tend to engage in health risk behaviors, further resulting in adverse health outcomes. Our research has confirmed that health risk behaviors play a mediating role of −0.074% in the impact of the social environment on adverse health outcomes. Specifically, health risk behaviors mediate −0.065% of the effect of the social environment on adverse health outcomes and −0.025% of the effect of family health on adverse health outcomes. From the perspective of social determinants theory, an individual’s social structure determines their life opportunities and socialization experiences, which, in turn, influence their life choices. Life opportunities and choices develop into habits through interactions, ultimately manifesting as behaviors related to healthy lifestyles (93). Therefore, it can be argued that social class or socioeconomic status is the fundamental determinant of health disparities, including health behaviors.

Going deeper, similar to the concept of the health-poverty trap, unstable employment populations may become ensnared in an adverse health outcome-social environment trap, potentially linked to the solidification of social stratification. The mechanisms of adverse health outcome-social environment trap are prominently characterized by a positive feedback loop: exposure to increasingly adverse social environments heightens the likelihood of adverse health outcomes, which, in turn, increase the probability of further exposure to even more adverse social environments. With the diminishing occurrence of “rags to riches” narratives (93), Chinese social structures have gradually solidified and social mobility has been decreased (94), resulting in a formidable “class divide” (95). Scholars like Stephens et al. (96) contend that social class is an ideal standard, behavioral norm, and social system shaped by society and history. Individual characteristics and social environmental characteristics can influence health behaviors and health outcomes by activating the cultural systems within social class. Therefore, individuals within the lowest strata of Chinese society face formidable challenges in breaking free from the adverse health outcome trap, much akin to their struggle to escape poverty traps or traverse social class boundaries.

## Conclusion

In the backdrop of the VUCA era, individuals with non-fixed employment in China face various uncertainties that make it challenging to avoid adverse health outcomes. However, this challenge is not a particular case in China but rather a critical research topic globally. Utilizing data from the *Psychology and Behavior Investigation of Chinese Residents 202*1 (PBICR-2021) and employing the PLS-SEM and fsQCA methods, we have explored the mediating mechanisms and complex configurations of the social environment, health cognition, and health behavior concerning adverse health outcomes among the unstable employment populations in China. Based on the PLS-SEM analysis, we made the following key findings. Social environment negatively influences adverse health outcomes, indicating that individuals in unstable employment have difficulty escaping external environmental control. Social environment has a positive impact on health cognition, making it difficult for the group improving health cognition. Social environment negatively affects health risk behaviors, and individuals in unstable employment struggle to change their behavioral habits. Furthermore, health risk behaviors and health cognition mediate the influence of the social environment on adverse health outcomes, underscoring the difficulty for unstable employment populations in breaking free from the adverse health outcome trap. In the fsQCA analysis, we found that adverse health outcomes among unstable employment populations result from multiple antecedent conditions in complex configurations. No single variable is sufficient to adequately explain their causal relationships. Weak social support emerges as a core condition leading to depression and anxiety outcomes, which are also attributed to multiple factors. Additionally, there are shared configurations and causal pathways between physical health impairment and depression outcomes, suggesting potential interconnections among multiple adverse health outcomes. Overall, our research aligns with the tenets of the social determinants of health theory, emphasizing that the fundamental cause of the entrenched adverse health outcomes among China’s unstable employment populations is health inequality stemming from class stratification as well as the uncertainties shock of external factors in the era of VUCA.

Theoretically, our research enriches and expands the viewpoints of the health social determinants theory, demonstrating the mechanism of adverse health outcomes among the individuals with Non-Fixed Employment, and constructs a theoretical model that is fully explanatory both in terms of linear relationships and multiple concurrent causal relationships. Practically, this study can provide a reference for the government to formulate policies to improve the adverse health outcomes of the individuals with Non-Fixed Employment better safeguard the health of the public. At the same time, it can also provide a basis for the individuals with non-fixed employment to maintain their own health levels to a certain extent.

### Limitations and future directions

However, this study has its limitations. We did not define the duration of unstable employment status, nor did we measure the health status of unstable employment populations before their transition to unstable employment, making it challenging to infer whether their poor health status contributed to their unstable employment status. Moreover, our study employed cross-sectional data, which cannot reveal the dynamic causal relationships between the variables under investigation. Given that the primary objective of our research was to explore the influencing factors and mechanisms of adverse health outcomes, and we have successfully drawn corresponding conclusions, addressing these limitations will be a focus of our future research efforts.

## Data Availability

The raw data supporting the conclusions of this article will be made available by the authors, without undue reservation.
